# Pyroptosis, superinfection, and the maintenance of the latent reservoir in HIV-1 infection

**DOI:** 10.1038/s41598-017-04130-9

**Published:** 2017-06-19

**Authors:** Dominik Wodarz, David N. Levy

**Affiliations:** 1 Department of Ecology and Evolutionary Biology & Department of Mathematics, 321 Steinhaus Hall, University of California, Irvine, CA 91697 USA; 2 Department of Basic Science, 921 Schwartz Building, New York University College of Dentistry, New York, NY 10010 USA

## Abstract

A long-lived reservoir of latently infected T cells prevents antiretroviral therapy from eliminating HIV-1 infection. Furthering our understanding of the dynamics of latency generation and maintenance is therefore vital to improve treatment outcome. Using mathematical models and experiments, we suggest that the death of latently infected cells brought about by pyroptosis, or to a lesser extent by superinfection, might be key mechanisms to account for the size and composition of the latent reservoir. Pyroptosis is a form of cell death that occurs in a resting (and thus latently infected) T cell when a productively infected cell attempts cell-to-cell transmission of virus. Superinfection of latently infected cells by productive virus could similarly remove those cells through active virus replication and resulting cytopathicity. The mathematical models presented can explain a number of previously published clinical observations including latent reservoir size and the relationships to viral load in acute HIV infection, measurements of the latent reservoir in chronic infection, and the replacement of wild-type virus by CTL escape mutants within the latent reservoir. Basic virus dynamics models of latency that do not take into account pyroptosis, superinfection, or other potential complexities cannot account for the data.

## Introduction

A major obstacle to eradication of HIV-1 from patients (i.e. a cure) is the presence of a reservoir of long-lived latently infected cells, of which resting memory CD4 T cells are the best characterized^1^. According to one study, the latent reservoir has an estimated half-life of about 6 months in patients who have been on anti-viral treatment for at least half a year and in whom there is no evidence of ongoing virus replication during therapy^2^. In other studies in which patients had been on anti-viral treatment for several years, the estimated half-life was much longer, about 31 or 42 months, respectively^3, 4^. These numbers indicate that current treatments are unable to deplete the latent reservoir during the life of an infected individual. In early attempts at developing eradication strategies, so-called shock and kill approaches have been used to activate latent HIV-1 during antiretroviral therapy^5^. The hope is that viral cytopathicity and/or immune responses will then allow elimination of the latent reservoir. While activation of some virus expression has been achieved^6^, this has so far not been translated into a reduction in reservoir size. A detailed understanding of the principles that govern the generation and persistence of the latently infected cell reservoir is vital to advance our ability to overcome this obstacle.

An aspect of HIV-1 that has not been discussed much in the context of viral latency is the infection or attempted infection of latently infected cells. Infection of cells with multiple copies of HIV-1 has been documented in a variety of settings^7–9^. The majority of the HIV-1 latent reservoir resides in resting CD4 T cells that, while historically have been viewed as refractory to HIV-1 infection, have more recently been shown both in vitro and in vivo to be permissive to infection, albeit with slower kinetics than activated T cells^10^. Productive superinfection of a latently infected cell would result in the elimination of the latent virus genome through cell death.  A similar effect, and perhaps more relevant, could arise from the induction of pyroptosis in the latently infected target cells during attempted direct cell-to-cell transmission through virological synapses^11^. It has been shown that attempted transmission of virus from a productively infected cell to a resting T cell can result in failure of transmission due to incomplete reverse transcription. The resulting partial DNA products of the virus trigger an innate immune response in the cell that leads to an inflammatory death process of the resting cell, called pyroptosis. This process has been implicated in the destruction of the resting T cell population during HIV-1 infection^12^. Induction of pyroptosis in latently infected resting T cells during attempted superinfection could thus also potentially impact the size of the latent virus reservoir. In principle, both pyroptosis (death due to attempted superinfection) and death due to actual superinfection of latently infected cells can affect dynamics in a very similar way, with the relative importance of the two mechanisms depending on the kinetic parameters. This paper examines the dynamics of generation and maintenance of the latent reservoir in the absence and presence of pyroptosis and superinfection of latently infected cells.

## Materials and Methods

Virus dynamics are modeled with ordinary differential equations and agent-based models that track the time at which individual latently infected cells were generated. Details are provided in the main text and Supplementary Materials. Experiments are based on previous work^7^, and are summarized in Supplementary Materials.

### Basic model

In the simplest form, the dynamics of latency generation in the context of multiple infection can be formulated by a system of three ordinary differential equations^13^. Denoting uninfected target cells by S, productively infected target cells by I, and latently infected target cells by I_0_, the equations are given as follows.

1 $$\begin{array}{c}\frac{{dS}}{{dt}}={\lambda }-{dS}-{\beta }SI\\ \frac{dI}{dt}=q\beta SI-aI+fq\gamma {I}_{0}I+g{I}_{0}\\ \frac{d{I}_{0}}{dt}=(1-q)\beta SI-{a}_{0}{I}_{0}-fq\gamma {I}_{0}I-\gamma {I}_{0}I-g{I}_{0}\end{array}$$

Susceptible, uninfected target cells are produced with a rate λ, die with a rate d, and become infected with a rate β. Assuming that the virus population is in a quasi-steady state^13^, this can be considered as a composite parameter of free virus and direct cell-to-cell transmission through virological synapses, such that β = β^free^ + β^syn^, where β^free^ and β^syn^ are the rate constants for the respective transmission pathways^14^. *In vitro* experiments have estimated both transmission pathways to contribute about equally to virus spread^14^.

Infection results in the generation of a productively infected cell with a probability q. Productively infected cells die with a rate a. With a probability 1-q, infection of uninfected target cells results in the generation of a latently infected cell. Latently infected cells die with a rate a_0_, and return to being productively infected with a relatively slow rate g. It is further assumed that latently infected cells can be successfully superinfected by productive virus, which is described by the term fqγ. Because latently infected cells are mostly resting T cells and because recent work suggests that those can only be viably infected through free virus transmission^10, 11^, we set γ = β/2^14^. Moreover, the productive infection rate of resting T cells is lower than that of activated cells^10^, and this is expressed by the parameter f < 1. Apart from superinfection of latently infected cells by productive virus, multiple infection of cells is not modeled.

If attempted infection of resting T cells, and thus latently infected cells, occurs through synaptic transmission, it has been shown that the resting T cells undergo a form of cell death called pyroptosis^10^. Hence, upon contact with a productively infected cell, pyroptosis of the latently infected cell occurs with a rate γ = β/2, using the estimate that cell-to-cell transmission contributes about half to virus spread *in vitro* ^14^. This modeling adds to previous mathematical work describing the process of pyroptosis^15^.

As outlined above, model assumptions are based on previously published data. As with any mathematical modeling study, however, it has to be remembered that results depend on assumptions and formulations, some of which remain uncertain. One aspect that has not been directly shown with data is that latently infected primary T cells *in vivo* can be superinfected. Superinfection of latently infected cells has only been shown in the context of cell lines *in vitro* ^16, 17^. On the other hand, resting CD4 T cells are directly infected at relevant frequencies *in vivo* ^17–19^, thus there is no a priori reason why a latently infected resting CD4 T cell should not be reinfected at a rate similar to infection of a previously uninfected cell.

This model is characterized by two equilibria that can potentially be stable. (i) The virus population is extinct and the target cell population is present at healthy levels. The equilibrium expressions are given by S^(0)^ = λ/d, I^(0)^ = 0, I_0_^(0)^ = 0. (ii) The virus successfully establishes an infection, leading to an equilibrium where all populations persist. This equilibrium is given by lengthy expressions that are solutions of second degree polynomials and are therefore not written down here. The virus infection persists if $\frac{\beta \lambda ({a}_{0}q+g)}{da({a}_{0}+g)} > 1$, obtained by stability analysis. It is unclear how this quantity relates to the basic reproductive ratio (R_0_) of HIV-1 that has been measured in patients^20^. Since those estimates have been performed using virus growth curves *in vivo*, they likely reflect only the contribution of productively infected cells. When choosing our parameter values to match R_0_ estimated in patients^20^ we will thus use R_0_ = (qβλ)/(da), which is the basic reproductive ratio of the virus in a corresponding model with only productive infection^13^. Model parameters used for computer simulations are discussed in the Supplementary Materials.

### Equilibrium properties

**Figure 1:**
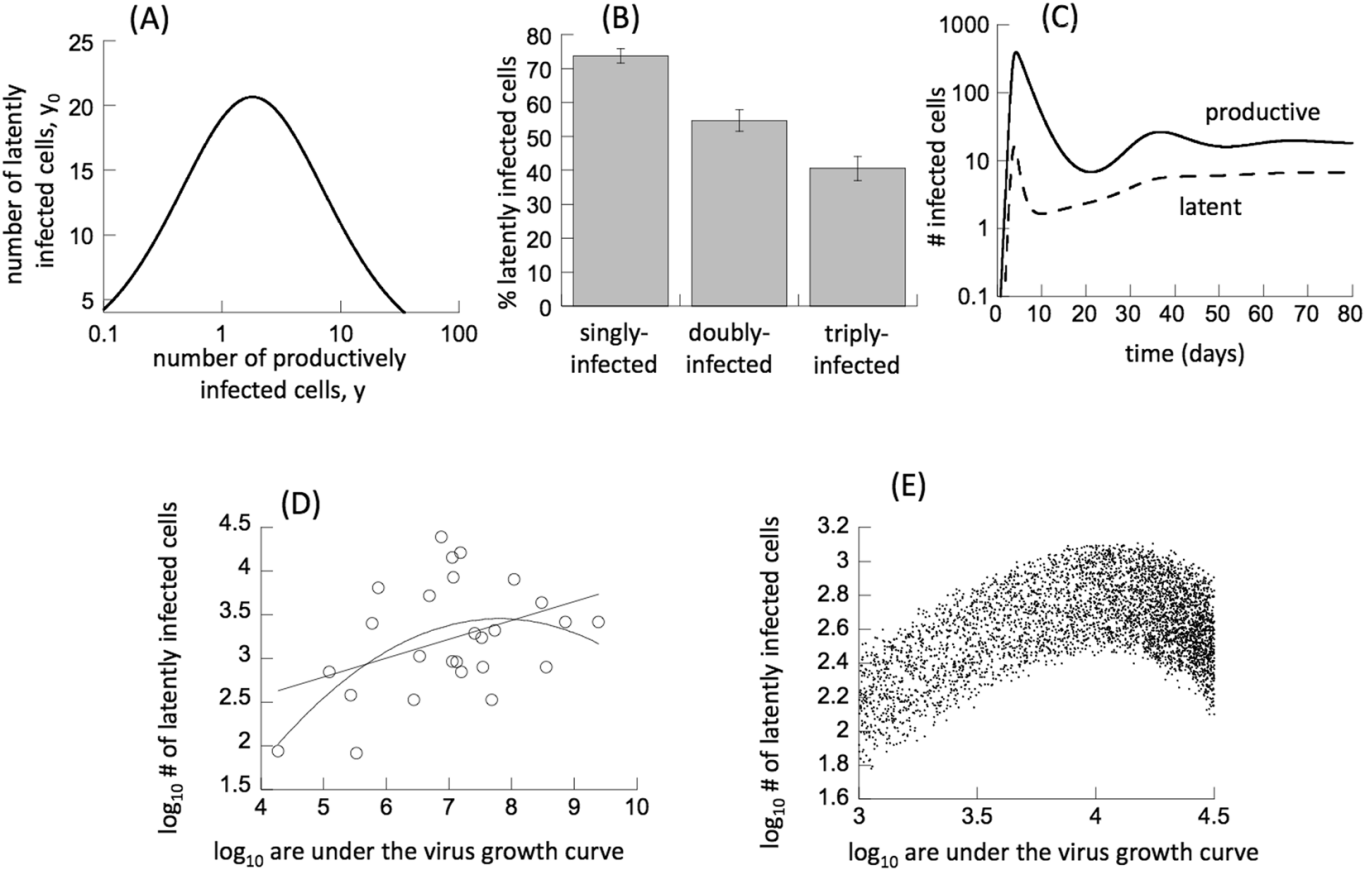
(**A**) Basic properties of model (1). The death rate of infected cells, a, was varied (from a = 0.1d^−1^ to a = 1d^−1^), and the relationship between the equilibrium number of latently and productively infected cells was plotted. λ = 10 d^−1^, d = 0.015 d^−1^, a_0_ = 0.003 d^−1^, g = 0.001 d^−1^, β = 0.0057/(dayxcells), q = 0.95, f = 1/7. Higher values of the death rate parameter *a* can be thought of representing a CTL response that reduces the life-span of infected cells. (**B**) *In vitro* latency following infection of Jurkat T cells with fluorescent reporter viruses. Plotted is the calculated percentage of latently infected cells (those without a productive virus) among all cells that are singly, doubly, and triply infected. Details of data and calculations are given in the Supplementary Materials. The average from six replicas of the experiment was determined, and standard errors are shown. According to t-tests, the difference was significant comparing the average percentage among singly and doubly infected cells (p = 0.039), among singly and triply infected cells (p = 0.00049), and among doubly and triply infected cells (p = 0.032). (**C**) Acute infection dynamics, predicted by model (1). λ = 10 d^−1^, d = 0.015 d^−1^, a = 0.45 d^−1^, a_0_ = 0.003 d^−1^, g = 0.001 d^−1^, β = 0.0057/(dayxcells), q = 0.95, f = 1/7. (**D**) Data replotted from reference 24: As in the original paper, a straight line was fitted through these data (p = 0.04). Also, a polynomial function can be fit through these data, describing a one-humped relationship (p = 0.006). The linear model has a slightly lower Akaike information criterion (AIC) value than the polynomial model, with a difference of only Δ = 0.38. (**E**) Relationship between the number of latently infected cells and the area under the virus growth curve, according to stochastic Gillespie simulations^36^ of model (1). For each realization of the simulation, parameters were chosen randomly from a uniform distribution, assuming a range of +/−10% of the base values. Each dot represents one realization. The time at which measures were determined was also randomly chosen in the range between 1 and 11 days. The base parameters are: λ = 100 d^−1^, d = 0.003 d^−1^, a = 0.45 d^−1^, a_0_ = 0.003 d^−1^, g = 0.001 d^−1^, β = 0.000114/(dayxcells), q = 0.95, f = 1/7. Parameters were adjusted compared to other figures to ensure persistence in the stochastic setting.

First, we consider virus persistence at equilibrium. In particular, we investigate how the number of latently infected cells correlates with the number of productively infected cells (directly related to viral load) at equilibrium. One of the main parameters that determines the equilibrium level of productively infected cells in this model is the death rate of infected cells, a. A higher death rate of infected cells results in less virus production during the life-time of infected cells in our model, and thus leads to a reduced rate of virus spread, consistent with previously published work^13^. In our model, the death rate parameter can in principle be caused both by virus cytopathic effects or by immune responses including the cytotoxic T lymphocyte (CTL) response, because immune responses are not explicitly included for now (see below for immune models). Thus, we varied the death rate of infected cells, *a*, in the model, and the correlation between the number of productively and latently infected cells at equilibrium is shown in Fig. 1A. We observe a one-humped relationship. At low virus loads, the number of latently infected cells is low, because less virus spread gives rise to fewer latently infected cells. At high virus loads, however, the number of latently infected cells is also low. The reason is that a relatively higher proportion of latently infected cells becomes either productively infected resulting in virus-induced cell death, or undergo pyroptosis upon attempted cell-to-cell transmission. This depletes the pool of latently infected cells at high virus loads. For intermediate virus loads, the number of latently infected cells is highest. There is enough virus spread to generate an abundance of latently infected cells, while only little pyroptosis or superinfection of those latently infected cells occurs.

### Infection multiplicity influences the latent pool: experimental data

Model (1) generated the hypothesis that superinfection of latently infected cells and induction of pyroptosis in latently infected cells might be important factors that influence the size of the latent reservoir. Both processes act in the same fashion: the (attempted) multiple infection of latently infected cells causes their death. We aimed to address this basic concept with *in vitro* experiments, using the infection of Jurkat T cells with fluorescently labeled HIV-1. In this experimental system, pyroptosis is unlikely to be a significant process. However, a significant amount of latently infected cells is generated in cell culture, and those cells can be readily superinfected^7, 21, 22^. We infected Jurkat T cells with equal amounts of three single round reporter viruses of different fluorescent color (GFP, YFP, and CFP)^7^, as described in the Supplementary Materials. Three days after infection, the percentage of cells fluorescent in each color or combination of colors, and the total number of fluorescent cells, was assessed by flow cytometry. The fluorescing infected cells correspond to productively infected cells, but it is not possible to determine infection multiplicity because a cell can be infected with multiple copies of a virus fluorescing in the same color. By assuming that infection follows a modified Poisson distribution, we can calculate the number of cells that are infected with 1, 2, or 3 viruses, at least one of which is productive. Details are provided in the Supplementary Materials. Similarly, we can calculate the number of cells infected with 1, 2, and 3 viruses, none of which is productive, corresponding to latently infected cells with a long life-span. The latter is of interest with respect to our theory, and plotted as a function of infection multiplicity in Fig. 1B. The calculated percentage of latently infected cells (infected cells that contain no productive viruses) among all infected cells was found to be significantly smaller among the population of doubly and triply infected cells, compared to the population of singly-infected cells. This supports our theoretical notion that multiple infection, which correlates with the extent to which latently infected cells are superinfected, can significantly impact the size of the latent reservoir, leading to its reduction through cell death.

### Modeling acute infection dynamics

Next, we consider the dynamics of acute infection, that is, a rise of virus load up to a peak, followed by a decline and convergence to an equilibrium. We are interested in how the population of latently infected cells develops over time as virus load rises and declines. This is explored with model (1) above and shown in Fig. 1C. At first, as virus load rises from low levels, the population of latently infected cells also increases. When virus load reaches relatively high levels and approaches its peak, however, the number of latently infected cells declines (Fig. 1C). The reason is that at high virus loads, a relatively large amount of pyroptosis/superinfection of latently infected cells occurs, resulting either in cell death caused by productive virus replication or in pyroptosis. As overall virus load declines and the system converges to an equilibrium, the population of latently infected cells rebounds and rises to a certain extent (Fig. 1C). This is because new latently infected cells are being generated by virus replication, while the lower virus loads ensure that only a small amount of pyroptosis/superinfection occurs, thus preserving the pool of latently infected cells.

Data from HIV-infected patients indicated that a smaller pool of latently infected cells was present during the acute phase than later in chronic infection, suggesting the accumulation of more latently infected cells as a function of time since transmission^23^. Our modeling results suggest an alternative interpretation: In our model, during the initial stages of virus growth, the number of latently infected cells correlates with virus load, while the relatively low number of latently infected cells found during the peak of acute infection is caused by high levels of pyroptosis/superinfection of latently infected cells during the period of high virus loads. In accordance with data, the model predicts that during chronic infection, when virus loads are lower, more latently infected cells are present. This is not because the virus has had more time to generate them, but because at lower virus loads during chronic infection, less pyroptosis/superinfection of latently infected cells preserves this population.

A previous study estimated the size of the latently infected cell population during acute infection among a cohort of patients^24^. The authors correlated this measure with virus load, specifically with the area under the virus growth curve, before therapy was initiated. A statistically significant positive correlation was observed (p = 0.04) and these data are replotted in Fig. 1D. Apart from fitting a straight line through these data, as was performed in ref. 24, it is also possible to fit a polynomial function as we do in Fig. 1D. The best fit (p = 0.006) is a one-humped curve, where the number of latently infected cells first rises with a larger area under the virus growth curve, but then declines. The Akaike information criterion (AIC) indicates that although the linear relationship cannot be rejected, the polynomial model can also be a relevant description of the data. While the AIC is lower for the linear model, the difference in AIC is only Δ = 0.38. The interpretation of this small difference is that the polynomial model is a reasonable alternative to the linear model. The one-humped relationship is consistent with our theory presented above. To demonstrate this, we simulated this kind of data set with a variant of model (1) (Fig. 1E). Each point corresponds to one “patient”, i.e. one simulation with parameters that were randomly chosen around defined values (see Fig. 1E for details). In addition, the time when the latently infected cell population size was determined during the acute phase of the infection was varied randomly in the same way. We find that the number of latently infected cells does not show a linear correlation with the area under the virus growth curve, but that it displays a one-humped relationship instead (Fig. 1E).

### Latency and immune responses in chronic infection

In the following section we explore the number of latently infected cells present during chronic infection and how this can depend on the strength of immune responses that regulate virus load. Particular emphasis is placed on the relative abundance of productively and latently infected cells, comparing model predictions with and without pyroptosis/superinfection of latently infected cells.

#### Model without pyroptosis/superinfection

Over the short term during chronic infection, the CD4 T cell population stays relatively constant; thus, we can simplify the models by assuming a c onstant overall CD4 T cell population. Moreover, immune responses limit virus load during chronic infection by both lytic (e.g. CTL) and non-lytic (antibodies and other inhibitory factors released by cells) mechanisms. Lytic immune responses eliminate productively infected cells, reducing virus output, while non-lytic responses inhibit new infection events. Taking into account these assumptions, the model without pyroptosis/superinfection can be formulated as follows^25^.

2 $$\begin{array}{c}\frac{dI}{dt}=\frac{q\beta SI}{{p}_{1}Z+1}-aI+g{I}_{0}-{p}_{2}IZ\\ \frac{d{I}_{0}}{dt}=\frac{(1-q)\beta SI}{{p}_{1}Z+1}-{a}_{0}{I}_{0}-g{I}_{0}\\ \frac{dZ}{dt}=cI-bZ\end{array}$$

where the susceptible cell population is given by *S* = 1 − *I* − *I*_0_. The anti-viral immune response is denoted by Z, and represents a generic adaptive immune response, without specifying the exact identity. This response can inhibit viral replication by non-lytic means with a rate p_1_, and can lyse productively infected cells with a rate p_2_^25^. This response can thus represent either CD8 T cell or B cell responses. The specific immune cell population, and thus the strength of this response, increases in reaction to antigenic stimulation with a rate c, and decays with a rate b in the absence of antigenic stimulation. The condition for infection establishment is identical to that of model (1). If an infection is successfully established, the system converges to an equilibrium where all populations persist.

First, we investigate the regime in which immune responses are non-lytic, i.e. p_2_ = 0. In this case, the equilibrium is given by the following expressions:

$$\begin{array}{c}{I}^{\ast }=\frac{b[\beta (q{a}_{0}+g)-a({a}_{0}+g)]}{b\beta a(1-q)+b\beta (q{a}_{0}+g)+{p}_{1}ca({a}_{0}+g)}\\ {I}_{0}^{\ast }=\frac{ab(1-q)[\beta (q{a}_{0}+g)-a({a}_{0}+g)]}{({a}_{0}q+g)[b\beta a(1-q)+b\beta (q{a}_{0}+g)+{p}_{1}ca({a}_{0}+g)]}\\ {Z}^{\ast }=\frac{c[\beta (q{a}_{0}+g)-a({a}_{0}+g)]}{b\beta a(1-q)+b\beta (q{a}_{0}+g)+{p}_{1}ca({a}_{0}+g)}.\end{array}$$

**Figure 2:**
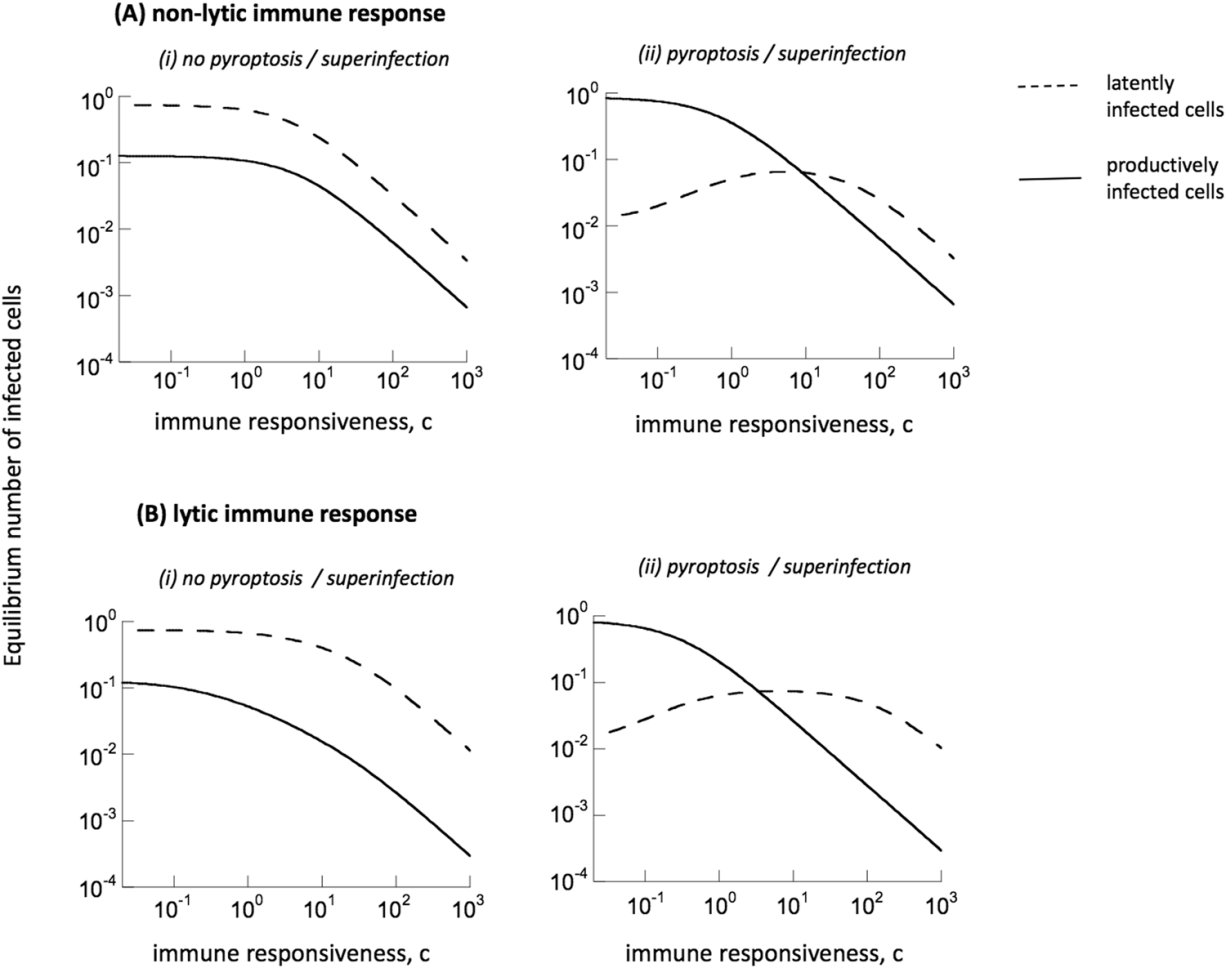
Exploring the relative equilibrium abundance of productively (solid line) and latently infected (dashed line) cells in chronic infection models (2/3). The graphs plot the equilibrium cell populations as a function of the immune responsiveness parameter c. Model (2) without pyroptosis/superinfection is compared to model (3) with pyroptosis/superinfection of latently infected cells. (**A**) The models assume a non-lytic immune response. Parameters were chosen as follows: a = 0.45 d^−1^, a_0_ = 0.003 d^−1^, g = 0.001 d^−1^, β = 3.6/(dayxcells), q = 0.95, p_1_ = 1/(dayxcells) b = 0.1 d^−1^, f = 1/7. (**B**) The models assume a lytic immune response. Parameters were chosen as follows: a = 0.45 d^−1^, a_0_ = 0.003 d^−1^, g = 0.001 d^−1^, β = 3.6/(dayxcells), q = 0.95, p_2_ = 1/(dayxcells), b = 0.1 d^−1^, f = 1/7.

We investigated the equilibrium number of latently infected cells relative to the number of productively infected cells in this model, as a function of the rate of immune cell expansion, c. This is because this parameter has a strong impact on equilibrium virus load in this model^13, 25^. Data indicate that latently infected cells make up about 4% of all infected T cells in untreated chronic infection^26^. In this part of the paper, the latently infected cells are assumed to die with a rate a_0_ = 0.003, and the latent virus within can become spontaneously active with a rate g = 0.001. This corresponds to the shorter measured half-life of the latently infected cell population (about 6 months). Other parameters are the same as discussed before. Immune parameters are set arbitrarily and presented results do not depend on this on a qualitative level. Figure 2Ai shows the equilibrium number of productively and latently infected cells as a function of the immune responsiveness parameter c. With higher immune responsiveness (larger c), both populations become lower. The important finding, however, is that the number of latently infected cells is always significantly larger than the number of productively infected cells, regardless of the immune responsiveness. This is clearly in contradiction to biological data^26^. In the model, latently infected cells are less abundant than productively infected cells if $\frac{{I}_{0}^{\ast }}{{I}^{\ast }}=\frac{(1-q)a}{{a}_{0}q+g} < 1$, i.e. if $q > \frac{a-g}{a+{a}_{0}}$. Because the death rate of productively infected cells, a, is most likely one or two orders of magnitude larger than the death rate, a_0_, or activation rate, g, of latently infected cells, this threshold for q is very close to one. Therefore, unless the probability for the virus to enter latency upon infection is very small (q very close to one), this model makes the unrealistic prediction that most infected T cells in the chronic phase will be latently infected. Even if the probability of productive infection, q, is sufficiently close to one such that there are more productively than latently infected cells, the value of q has to be even closer to one in order to obtain the prediction that latently infected cells make up only about 4% of all infected T cells, as observed in patients^26^. *In vitro* estimates from single-round HIV infection of cell lines reported latency occurrence in about 5–15% of infection events^16^, which is in contrast to the very low latency generation probability required in our current model to yield realistic fractions of latently infected cells. This measure of course needs to be still examined *in vivo*, but it can be argued that latency generation in cell lines is less likely than in primary T cells because cell lines do not enter resting states.

We previously mentioned that the infection rate β can be considered a composite of free virus and synaptic virus transmission. Synaptic transmission has been associated with the transfer of multiple viruses from one cell to another, which can lead to multiple infection of the target cell, and which can thus lead to a high probability that at least one of the these viruses will be productive. We re-did the above calculation assuming that latency can be generated with a probability q upon free virus infection, but that at least one virus always becomes productive upon synaptic transmission. While this reduces the latent pool to a certain extent, the above conclusions remain essentially unchanged (Supplementary Information).

Going back to the original model formulation, similar results are obtained if we assume that all immune activity is lytic (p_1_ = 0, p_2_ > 0). The equilibrium number of latently and productively infected cells as a function of the immune responsiveness, c, are plotted in Fig. 2Bi (the equilibrium expression are very lengthy and not written down here). As before, the majority of the infected T cells in this model are latently infected. We can again define the ratio $\frac{{I}_{0}^{\ast }}{{I}^{\ast }}=\frac{pc(\beta -{a}_{0}-g)+2b\beta (a-g)}{\beta [2b({a}_{0}+a)+pc]}$. From this, it follows that the equilibrium number of productively infected cells only exceeds the number of latently infected cells if the probability of productive infection, q, crosses a threshold, defined by

$q > \frac{pc(\beta -{a}_{0}-g)+2\beta b(a-g)}{\beta [2b({a}_{0}+a)+pc]}$. Under the realistic assumption that a ≫ a_0_ + g, and hence β ≫ a_0_ + g, this expression converges to one. Thus, again, in order to predict that latently infected cells only make up a small fraction of all infected T cells, the value of q has to lie very close to one, which means that the probability for a virus to become latent upon infection must be very small.

Note that we chose the larger of the two measured decay rates of the latent reservoir^2, 3^ for the computational simulations, because this results in a lower abundance of the latent reservoir. The slower estimated decay rate of the latent reservoir might be more realistic, and would strengthen the results reported in this section.

#### Model with pyroptosis/superinfection

Here, the above analysis is repeated assuming that latently infected cells can become superinfected with productive virus (resulting in its elimination) and that attempted superinfection of latently infected cells through virological synapses can result in pyroptosis of the latently infected cell. The above model can thus be re-written as follows.

3 $$\begin{array}{c}\frac{dI}{dt}=\frac{q\beta SI}{{p}_{1}Z+1}-aI+g{I}_{0}+\frac{fq\gamma {I}_{0}I}{{p}_{1}Z+1}-{p}_{2}IZ\\ \frac{d{I}_{0}}{dt}=\frac{(1-q)\beta SI}{{p}_{1}Z+1}-{a}_{0}{I}_{0}-g{I}_{0}-\frac{fq\gamma {I}_{0}I}{{p}_{1}Z+1}-\frac{\gamma {I}_{0}I}{{p}_{1}Z+1}\\ \frac{dZ}{dt}=cI-bZ\end{array}$$

Since the total target cell population is assumed to be constant, we again have S = 1-I-I_0_. Pyroptosis and superinfection of latently infected cells is described in the same way as in model (1), with the addition that both processes are inhibited by non-lytic immunity (both are related to infection processes).This model is analytically intractable, so equilibrium values were determined numerically in the following analysis. Similar patterns are observed for both lytic and non-lytic immune activity (Fig. 2Aii and Bii). First, we note that in contrast to the model without pyroptosis/superinfection, there is now a wide parameter region in which the latently infected cells are much less abundant than the productively infected T cells. The relative abundance of these two populations depends on the immune responsiveness, c (Fig. 2Aii and Bii). If the immune responsiveness is strong and the value of c lies above a threshold, then the number of productively infected cells is very low, and there are more latently infected cells than productively infected cells. This corresponds to a scenario where the infection is controlled very efficiently in the chronic phase, resulting in extremely low virus loads. Latently infected cells are not recognized by the immune response and therefore persist. As the immune responsiveness is lowered, the number of productively infected cells rises, and so does the number of latently infected cells. This is because higher virus load leads to the generation of more latently infected cells. When the immune responsiveness, c, falls below a threshold, the productively infected T cell population becomes dominant compared to the number of latently infected cells (Fig. 2Aii and Bii). The reason is that in this regime, virus load is higher, leading to the occurrence of more pyroptosis/superinfection. This leads to the death of more latently infected cells. This is also reflected by the observation that a further reduction in the immune responsiveness and the consequently higher number of productively infected cells leads to a reduction in the number of latently infected cells. More productively infected cells means more pyroptosis/superinfection of latently infected cells, which in turn translates into a lower number of latently infected cells.

This analysis has shown that in contrast to the model without pyroptosis/superinfection, the model with pyroptosis/superinfection can easily account for the clinical observation that the latently infected T cell pool constitutes only a small percentage of the overall population of infected T cells.

### Latency and the archiving of viral genomes

Persistent viral latency in long-lived cells, in combination with ongoing virus evolution, means that the latent reservoir represents an archive of genotypes that were prevalent at earlier stages of infection^27^. In contrast to this notion, recent data^28^ observed replacement of the latent reservoir with CTL-resistant virus variants during chronic infection, while during the earlier stages of infection, the latent reservoir had been largely made up of CTL-sensitive viruses predominant in this phase. The reasons for this almost complete replacement of viruses in the latent reservoir remains unexplained.

Here, we model the persistence of archival forms in the context of pyroptosis and superinfection. To do so, we construct a stochastic agent-based model that tracks individual virus genomes in individual cells over time, and also includes immune responses. This allows us to record the time when a viral genome becomes part of the latent reservoir and thus document the kinetics of viral archiving and removal from the virus pool. The model is described in Supplementary Information.

The computer simulations were started with populations around equilibrium levels, and was run for a duration of 1000 days. At the end of the simulation, the distribution of times at which existing latent virus genomes were generated was determined. The latent genomes were only counted in cells that did not contain productive virus, because this represents the latent reservoir that is relevant for virus persistence. Parameters were chosen as previously described for the ODE models. Simulations were run for both the shorter and longer half-life of the latent cell population estimated in the literature^2, 3^. We compared a strong response resulting in low numbers of infected cells with a weak response resulting in higher virus load. This was achieved by varying the immune parameters C and F (Supplementary Information).

**Figure 3:**
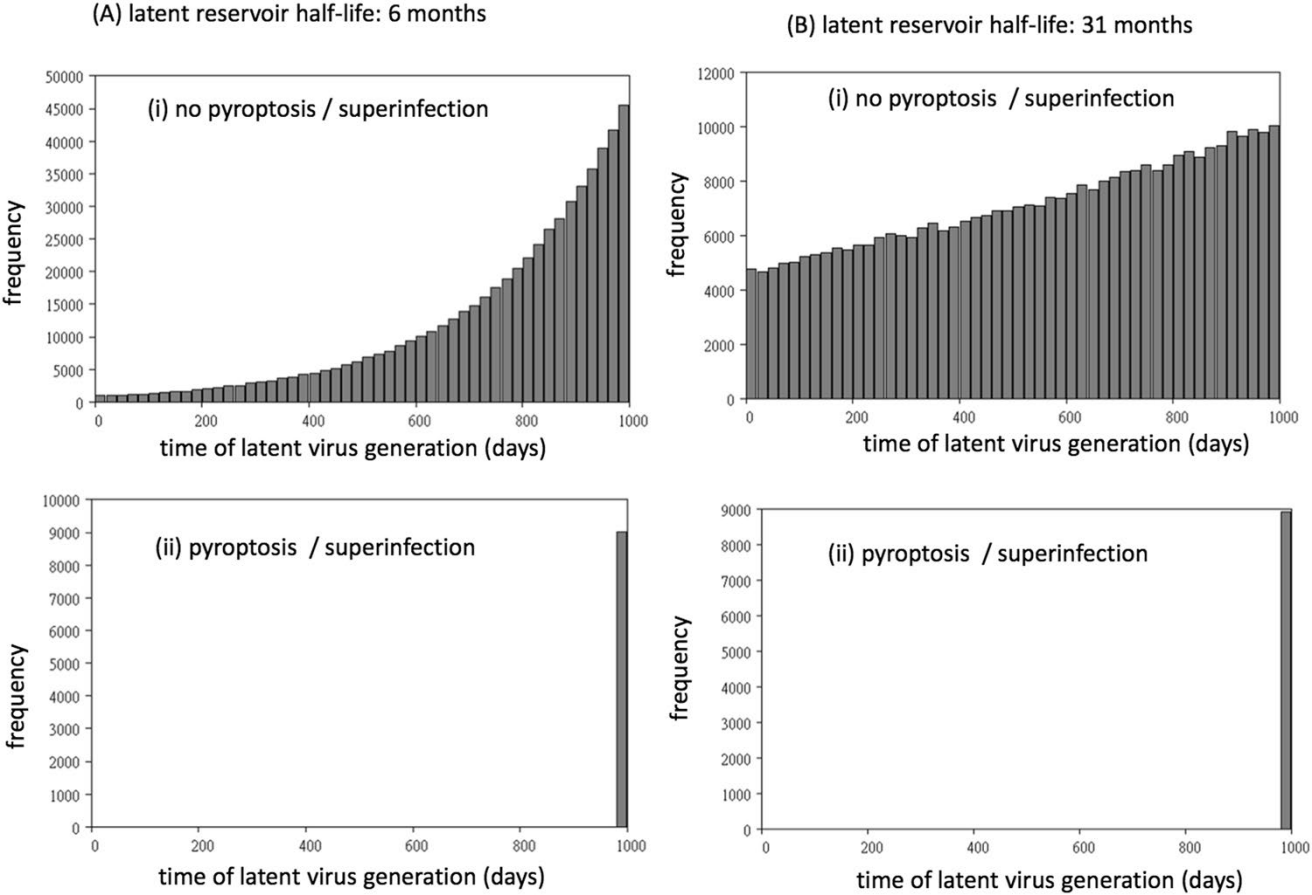
Archiving of viral genomes in the agent-based model assuming a relatively weak immune response against the virus. Computer simulations with and without pyroptosis/superinfection of latently infected cells are compared. The simulation tracks the time when latent genomes are generated during the course of the in silico infection, and the graphs are frequency distributions of the creation times of all latent virus genomes that are present at 1000 days post infection (in the latent reservoir, i.e. in cells that do not contain productive virus). (**A**) It is assumed that the latent reservoir has a half-life of about 6 months^2^. Each time step of the simulation corresponds to 0.1 days, and the probabilities per time step are given as follows. A = 0.045, A_0_ = 0.0003, G = 0.0001, B = 0.36, H = 0.01. During infection, Q = 0.95 is the probability of productive infection. The probabilities of CTL proliferation and CTL-induced inhibition of virus replication are determined by parameters C = 0.5, and F = 0.1, respectively. (**B**) Same simulation, assuming that the latent reservoir has a half-life of about 31 months^3^. Parameters were the same, except A_0_ = 0.00006, G = 0.000015.

**Figure 4:**
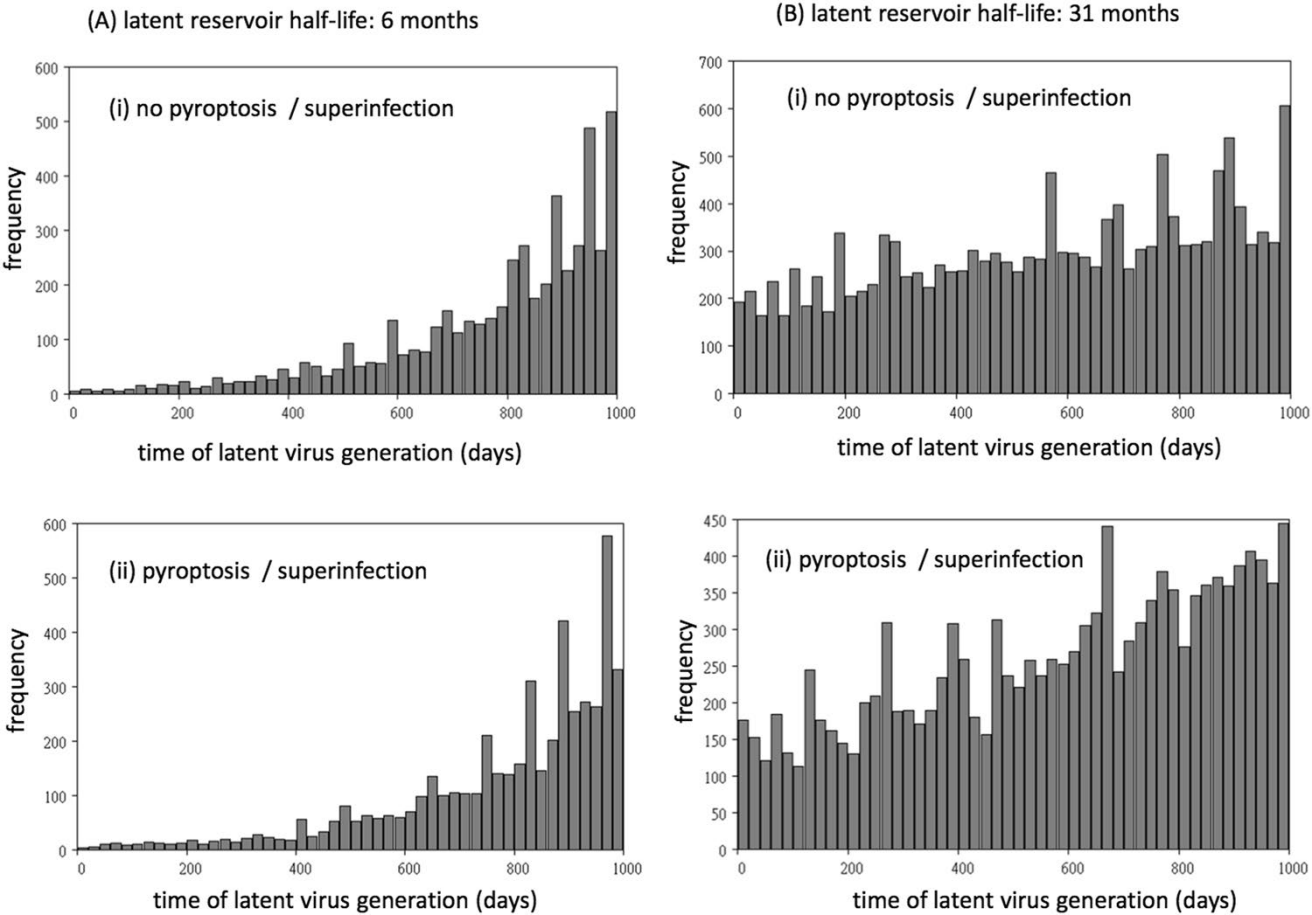
Influence of a stronger immune response on the archiving of viral genomes. Computer simulation was the same as in Fig. 3, except for the two parameters C = 10 and F = 10, which simulate a stronger immune response.

We compared simulations where pyroptosis and superinfection of latently infected cells did and did not occur. First consider a regime where the immune response is relatively weak (and thus virus load was relatively high, lower values of C and F in Supplementary Information, see Fig. 3). In simulations without pyroptosis and superinfection of latently infected cells, we observed that a significant amount of viral archiving occurred in the pool of latently infected cells (Fig. 3Ai,Bi). That is, viral genomes were present that had been generated at significantly earlier time points. As expected, this effect was more pronounced for the longer half-life of the latently infected cell population. In contrast, in simulations with pyroptosis and superinfection of latently infected cells, almost no archiving was observed, even for the longer half-life of the latent reservoir (Fig. 3Aii,Bii). That is, there was rapid replacement of the archived genomes with replicating viruses so that the latent cell reservoir contained only viral genomes that infected the cells very recently. The reason is that at higher virus loads, a significant amount of pyroptosis/superinfection of latently infected cells occurs, resulting in the elimination of the latent viral genomes that have entered the cells previously. This difference between simulations with and without pyroptosis/superinfection was greatly diminished if we assumed a stronger immune response that resulted in much lower virus loads (Fig. 4). In this case, significant viral archiving is still observed even in the presence of pyroptosis/superinfection. This makes sense, because less pyroptosis/superinfection occurs at lower virus loads that are maintained by the stronger immune response. With less pyroptosis/superinfection, the previously generated latent virus genomes are depleted to a lesser extent, allowing for more archiving.

These results reveal that in the presence of pyroptosis/superinfection of latently infected cells, the degree to which viral genomes are archived in the latent cell reservoir can depend on the strength of immune responses and the level of virus load. If virus load is relatively high, little archiving occurs. If virus load is suppressed to lower levels, more archiving occurs. During the long-term course of chronic HIV infection, virus load is at first relatively low and over time can increase due to a variety of factors, such as immune escape. This leads to the model prediction that the degree of viral archiving can become less pronounced over time. Particularly, events such as immune escape can lead to an increase in virus load, and this can lead to a deletion of the previously maintained viral archive, and the dominance of the recent escape mutants in the latent reservoir. As mentioned before, a dominance of CTL escape mutants in the latent reservoir has been demonstrated in clinical data^28^, and our theory provides a plausible explanation. This is demonstrated specifically in the Supplementary Information, using an extension of our agent-based model (see Figure S1).

## Discussion and Conclusion

Our work suggests that pyroptosis and/or the superinfection by productive virus of latently infected cells might be an important force that determines observed patterns of viral latency in HIV-1 infection, including the size of the latent reservoir in the acute and chronic phases of infection, and the replacement of CTL-sensitive with CTL-resistant virus in the latent reservoir. Both mechanisms (pyroptosis and productive superinfection of latently infected cells) act in the same way and are to a first approximation interchangeable with respect to the aspects studied here. It is likely that pyroptosis is the dominant mechanism that drives the dynamics, because the productive infection of latently infected cells is thought to be a relatively unlikely event, although an appropriate cytokine environment can significantly promote this process^10^. The concept that resting T cells are not infectible arises from studies utilizing blood-derived cells, which are highly quiescent and refractory to infection^29^. However, HIV-1 replicates in lymphoid and mucosal tissues, where an abundance of factors such as cytokines, chemokines and stromal cells induce a higher level of permissiveness to HIV replication^30^. Further experiments remain to be done to quantitatively investigate the importance of both processes for shaping the dynamics and composition of the latent reservoir.

However, even if neither pyroptosis nor superinfection of latently infected cells is eventually shown to occur at sufficient levels to impact the size and composition of the latent reservoir in HIV-1 infection, our model suggests that other, yet to be identified, mechanisms must likely be invoked to explain experimental and clinical data. Basic infection models without additional complexities, such as model (2), have difficulty to account for the data discussed here. Even if pyroptosis/superinfection are shown to be significant factors that influence the dynamics of latency, additional mechanisms might very well further make contributions (e.g. abortative infections, a higher activation status of cells due to high virus load, or a carrying capacity of the latent reservoir^31^). More work needs to be done to investigate the forces that shape the size and composition of the latent virus reservoir in HIV-1 infection.

Any modeling result needs to be considered in the context of the assumptions that underlie the equations and the associated uncertainties (similar to experimental results depending on the underlying methodologies). Thus, we would like to highlight some uncertainties that are relevant in our models. (i) Latency generation: The prevailing model of latency envisions infection of an activated T cells immediately before entry into quiescence^26^. This model has virtue of concordance with the observation that most latently infected cells are memory T cells that have been previously activated^32^. Our model is consistent with this mechanism. A higher degree of complexity in the pathways associated with the generation of latent viral genomes, however, has recently been demonstrated^10^, and could potentially result in the need for more complex modeling approaches. (ii) Latency maintenance: Our model assumed that in the absence of pyroptosis and superinfection, the maintenance and decline rate of the latent virus reservoir is governed by the basic death rate of latently infected cells, and their activation rate. More complex processes, however, might be at work^31, 33^. An emerging literature^31, 32, 34^ argues that additionally, homeostatic proliferative mechanisms in the memory T cell compartment can also contribute to this process. This includes the notion of a “carrying capacity” for the latent reservoir^31^, which by itself might account for a limited size of the latent reservoir. While the effect of proliferative processes can potentially be captured in our model through a reduction of the death rate parameter, these concepts can be explicitly incorporated into the models discussed here, should they turn out to be dominant drivers of the dynamics. (iii) Immune responses: The dynamics of antiviral immune responses have been studied here in the context of minimally parameterized equations that have been extensively used in the literature^13, 25^. Differences in the formulation of the terms that characterize immune expansion can potentially result in different dynamics^35^. On the level of our investigation, however, this is unlikely to change results. We varied parameters that determine the strength of the immune response, which in turn have a major impact on the level of virus load at equilibrium. This has been shown to be a robust feature across many different immune responses models^13, 35^, thus indicating that the results reported here are robust in this respect.

## Electronic supplementary material

Supplementary Information
