# Supplementary material for: Pyroptosis, superinfection, and the maintenance of the latent reservoir in HIV-1 infection
Source: Sci Rep. 2017 Jun 19;7:3834. doi: 10.1038/s41598-017-04130-9 (PMC5476677; doi:10.1038/s41598-017-04130-9)
Supplement: Supplementary file 1 — Supplementary Information [file 41598_2017_4130_MOESM1_ESM.pdf]

Supplementary Information

Pyroptosis, superinfection, and the maintenance of the latent reservoir in HIV-1 infection

Dominik Wodarz and David N. Levy

1. Experimental Methodology

In vitro infection: Jurkat (Clone E6, ATCC) cells were infected with equal amounts of three VSV-G pseudotyped env-defective reporter viruses that are limited to a single round of infection: NLENG1-ES-IRES, NLENY1-ES-IRES and NLENC1-ES-IRES [1, 2]. These viruses differ only in the reporter gene they contain, either enhanced Green Fluorescent Protein ("GFP", Clontech) or the GFP derivatives, enhanced Yellow Fluorescent Protein (YFP) or enhanced Cyan Fluorescent Protein (CFP) and thus have equal replicative properties. Six dilutions of viruses were used to infect $2x10^6$ cells (Table S1). Flow analysis was performed on a FACStar Plus as previously described [1], and depending on virus dose, between 25,000 to 615,000 cells were analyzed per condition. Three days after the infection, the percentage of cells that showed fluorescence in one, two, or three colors was determined, as was the percentage all fluorescent cells (regardless of colors).

These data are shown in Table S1 for the different dilutions. Because an infected cell that fluoresces in one color can be infected with multiple copies of the same colored virus, we have to calculate the number of cells that are productively and latently infected with 1, 2, and 3 viruses. The generated data allow us to perform these calculations, as described in detail in Section 2. The average infection multicity and the probability for a virus to become latent upon

infection were calculated for each dilution (Table S1). These estimates were used to calculate the percentage of cells that are infected with 1, 2, and 3 viruses with at last one virus being productive (which we will refer to as "productively infected cells"). In addition, we calculated the percentage of cells that are infected with 1, 2, and 3 viruses, all of which are latent (which we will refer to as "latently infected cells"). The results are summarized in Table S1. The last column of Table S1 shows the percentage of latently infected cells among all infected cells containing 1, 2, and 3 viruses. This is the quantity we are interested in to address the theoretical prediction that pyroptosis and superinfection of latently infected cells reduces the size of the latent reservoir. The average of those values over the six repeats is plotted in Figure 1B. This demonstrates that the percentage of latently infected cells significantly decreases among multiply infected cells (containing 2 and 3 viruses), presumably due to the frequent superinfection of latently infected cells by productive virus (which leads to the death of the cells through virus replication).

Dilution (Inoc. MOI)	Data Collected: % cells fluorescing in n colors				Parameters		% cells with n viruses, with at least 1 productive virus			% cells with n latent viruses			% latently infected cells among all infected cells with n viruses		
	any	n=1	n=2	n=3	Avg. Mult.	P(latent)	n=1	n=2	n-3	n=1	n=2	n=4	n=1	n=2	n=3
32	4.009	3.629	0.363	0.018	0.228	0.821	3.257	0.676	0.070	14.900	1.394	0.087	82.062	67.341	55.261
16	14.079	10.275	3.347	0.457	0.669	0.773	7.774	4.609	1.374	26.489	6.848	1.180	77.310	59.769	46.208
8	23.843	14.760	7.490	1.593	1.025	0.734	9.770	8.686	3.892	27.006	10.166	2.551	73.433	53.924	39.598
4	41.763	17.738	16.605	7.420	1.887	0.713	8.196	13.247	10.804	20.403	13.730	6.160	71.342	50.896	36.310
2	57.555	17.359	23.216	16.980	2.720	0.685	5.647	12.938	14.995	12.275	11.432	7.098	68.490	46.909	32.128
1	79.234	8.424	22.226	48.584	5.194	0.697	0.872	3.844	8.563	2.010	3.640	4.395	69.739	48.636	33.918

Table S1. HIV latency in a serial dilution of HIV-1. The yellow area shows the experimental data, i.e. the percentage of cells that fluoresced in any color, and the percentage of cells that fluoresced in 1, 2, or 3 colors. These data can be used to calculate the average number of viruses per cell (Avg. Mult.) and the probability that a virus becomes latent upon infection (P(latent)), shown in the gray area of the table. These estimates are in turn used to calculate the percentage of cells that are infected with 1, 2, and 3 viruses, at least one of which is productive (green area). Similarly, we can calculate the percentage of cells that are infected with 1, 2, and 3 viruses, all of which are latent (blue area). Finally, shown in orange is the percentage of latently infected cells among infected cells containing 1, 2, and 3 viruses. The average of those percentages over the six replicas is plotted in Figure 1B.

2. Calculating the number of cells infected with 1, 2 and 3 viruses from the color experiments

The infection with different colors cannot directly yield an estimate for how many productive viruses are found in cells because cells can be infected with multiple copies of a virus with identical color. However, the true distribution of the number of productive viruses per cell can be calculated by assuming that infection follows a modified Poisson distribution. We start by assuming the absence of viral latency. If *m* is the mean multiplicity of infection, then the probability of having multiplicity *n* is given by $P(n)=m^n e^{-m}/n!$. The expected frequency of infected cells is $y=1-e^{-m}$.

Suppose now that for each virus that infects a cell, the probability to become latent and not glow is p. If however at least one virus in a cell establishes a productive infection, then it is assumed that all resident viruses are activated and glow [3]. If a cell is infected with n viruses, it has probability $1-p^n$ to glow. Therefore, the probability for a cell to be infected with n viruses and glow is given by

$$Q_{inf} = \sum_{n=1}^{\infty} P(n)(1-p^n) = 1-e^{-m(1-p)}$$

We need to calculate the probability for a cell to be infected with all three colors. In the presence of three colors, each virus is of a given color with probability 1/3. If a given cell is infected with n viruses (and glows), then all three colors are represented with probability $R_3=1-(3(2/3)^n-(1/3)^{n-1})$. The probability for a cell to show all three colors is given by

$Q_3 = \sum_{n=1}^{\infty} P(n)(1-p^n)R_3 = e^{-m}\left((e^{m/3}-1)^3-(e^{mp/3}-1)^3\right)$. If p=0, we have simply $e^{-m}(e^{m/3}-1)^3$. To

calculate the probability for a cell to display any two (but not three) colors, we note that if a cell contains n viruses (and glows), then any two colors are represented with probability $R_2=3(2/3)^n-6(1/3)^n$. The probability for a cell to show any two (but not three) colors is thus given by $Q_2=\sum_{n=1}^{\infty}P(n)(1-p^n)R_2=3e^{-m}\left((e^{m/3}-1)^2-(e^{mp/3}-1)^2\right)$. If p=0, this simplifies to $3e^{-m}(e^{m/3}-1)^2$.

The parameters m and p can be deduced from simultaneously solving the equations for e.g. Q_{inf} and Q_3, the values of which are known from the experiments. Then, the probability for a cell to be productively infected with n viruses is given by $P(n)(1-p^n)$. The probability to be infected with n latent viruses is given by $P(n)p^n$.

3. Model parameters used in computer simulations

Regarding model parameters, some are known and we used those in computer simulations. Thus, the death rate of productively infected cells is set to a=0.45 [4]. The decline rate of the latent reservoir is given by a_0+g and both the shorter half-life of 6 months [5] and the longer half-life of 31 months [6] will be used in different simulations. In doing so, it is assumed that the dominant component of this decline is the death of latently infected cells, a_0, rather than their activation, g, as defined in the appropriate figure legends. No data exist for more accurate assignment of these values, but this appears intuitively reasonable. Results do not change qualitatively if this assumption is reversed. Other model parameters are unknown, and their biological meaning can also depend on the way in which models are formulated. The unknown parameters will be set such that in the context of the model under consideration, the basic reproductive ratio of the virus corresponds to the measured value of $R_0 \approx 8$ [7]. For different model formulations, different parameter combinations fulfill this condition, explaining variations

in parameter values in numerical simulations of different models. We note that this value of R_0 has been measured in acute infection and might change during chronic infection, for which we currently lack precise estimates. We further assume in simulations that the productive infection rate of latently infected (resting) cells is reduced seven-fold compared to the productive infection rate of the susceptible cell population S, which most likely are activated (i.e. $f=1/7$). It has been shown that the productive infection rate of resting T cells can vary a lot, depending on the exact conditions experienced by the resting T cells [8]. In the absence of cytokines, the productive infection rate of resting T cells is very much reduced compared to that of activated cells, on the order of 10-50 fold. In the presence of appropriate cytokines, however, the productive infection of resting T cells becomes much more likely, depending on the nature of the cytokines that are present. The chosen value of the parameter f is consistent with experimental observations if T cells are exposed to cytokines [8]. The pyroptosis rate of latently infected cells was set to 0.5β, i.e. half of the overall infection rate of susceptible cells. The rational for this is that the overall infection rate is composed of both infection via free virus and directly from cell to cell through virological synapses. Previous work has shown that synaptic transmission contributes approximately half to the overall rate of virus spread *in vitro* [9]. Since the rate of pyroptosis is determined by the rate of synapse formation, and since we lack information about the relative importance of synaptic transmission to virus spread *in vivo*, this is the assumption that is most consistent with available data. Finally, we do not have a good estimate for the probability of the virus to become productive / latent upon infection. Throughout the paper, we assumed that productive infection is established with a probability $q=0.95$.

4. Model assuming no latency generation during synaptic transmission

Model (2) in the main text suggests that in the absence of pyroptosis and superinfection of latently infected cells, the population of latently infected cells is dominant over the productively infected cell population, unless the probability of productive infection is very close to one. Therefore, the model has difficulty to account for the observation that latently infected cells only make up a rather small fraction of the total infected cell population. In model (2), we assumed that all infection events resulted in productive infection with a probability q, and in a latent virus with a probability 1-q. As explained in the main text, however, the infection rate can be considered a composite of free virus and synaptic transmission, with each pathway contributing equally to virus spread at least in vitro [9]. If multiple viruses are transferred per synapse, and if this leads to the multiple infection of the target cell, then the probability that one of those viruses becomes productive might be close to one. This in turn can potentially reduce the size of the latent reservoir. We investigated whether this could impact our conclusions, assuming the extreme case where upon synaptic transmission, at least one virus always ends up productive. With this assumption, model (2) can be re-written as follows.

$$\frac{dI}{dt} = \frac{q\gamma SI}{p_1 Z + 1} + \frac{\gamma SI}{p_1 Z + 1} - aI + gI_0 - p_2 IZ$$

$$\frac{dI_0}{dt} = \frac{(1-q)\gamma SI}{p_1 Z + 1} - a_0 I_0 - gI_0 \qquad (2)$$

$$\frac{dZ}{dt} = cI - bZ$$

where γ= β/2 [9]. For this model, the condition for there to be fewer latently than productively infected cells becomes $q > \frac{a - a_0 - 2g}{a + a_0}$, which is again very close to one. Whether this formulation is more realistic than model (2) remains open to investigation. While multiple viruses are clearly transferred through virological synapses [10, 11], it is less clear whether many of them successfully integrate into the genome of the target cell. The prevalence of multiple infection in vivo is currently debated in the literature [12-14].

5. Agent-based model describing the archiving of viral genomes

Here, we model the persistence of archival forms of the virus in the context of pyroptosis and superinfection of latently infected cells. To do so, we construct a stochastic agent-based model that tracks individual virus genomes in individual cells over time. This allows us to record the time when a viral genome becomes part of the latent reservoir and thus to document the kinetics of viral archiving and removal from the virus pool. As in the chronic infection models considered in the main text, a constant target cell population is assumed here as well. The model contains N cells, which can be either infected or uninfected. Two types of infected cells are distinguished: those that contain only latent virus (the latent reservoir), and those that contain at least one productive virus. While previous models did not consider multiple infection in cases other than pyroptosis / superinfection, the current model keeps track of all viral genomes in cells. At each time step, the system is sampled N times. The following rules are then applied. If the sampled cell is productively infected, the following events can happen. (i) The cell can die with a probability A. In this case, the dead infected cell is immediately replaced by an uninfected cell

(in order to maintain the constant target cell population). (ii) With a probability B, the cell attempts to transmit a virus to a target cell that is randomly chosen from the whole system. If the target cell is uninfected, the attempted infection event is always successful. In this case, the target cell becomes productively infected with a probability Q, and latently infected with a probability 1-Q. If the target cell is already productively infected, the same principles apply and the infection multiplicity is increased. With a probability Q, the new virus becomes productive, and with a probability 1-Q, the new virus becomes latent. In either case, however, the cell remains productively infected (because it already contains productive virus). Note that in this model, the rate of virus output from an infected cell does not depend on its multiplicity. If the chosen target cell is latently infected, on the other hand, the attempted infection event is not always successful. With probability one half, the attempted infection event is assumed to occur through virological synapses, resulting in cell death of the target cell by pyroptosis. If that does not occur (also probability one half), the attempted infection is assumed to occur through free virus transmission. Now, the following rules apply. With a probability Q/7, productive infection is established in the latently infected target cell, essentially turning the cell productive. With a probability (1-Q)/2, the incoming infection is also latent, leaving the infected cell in a latent state. These probabilities for establishing productive and latent infections in latently infected target cells are consistent with observed infection frequencies of resting T cells in the presence of appropriate cytokines [8]. With a probability 1-Q/7-(1-Q)/2, the attempted free virus infection event is unsuccessful and nothing happens. With our chosen parameters, this is the most common outcome with a probability of 0.84. If the sampled cell is latently infected, it can (i) die with a probability A_0, and is immediately replaced by an uninfected cell. (ii) With a probability G,

the latent virus becomes spontaneously active, and this turns the latently infected cell into a productively infected cell.

So far, this model describes the dynamics of the virus. Because we are modeling chronic infection, however, we also need to take into account adaptive immune responses, as in the previous model. Again, we consider a “generic” adaptive immune response. The number of immune cells increases as the immune response grows stronger with increasing virus loads, and grows weaker (fewer immune cells) as virus load declines. In the simulations, it is assumed that there is no competition for space between target cells and immune cells. We assume that there can be maximally M immune cells present in the system; in other words, our system contains M spots that are separate from the target cell spots. These spots can either be empty or occupied with an immune cell. At each time step, the system is sampled K times, where K is the number of immune cells that are currently present. If the sampled spot is empty, nothing happens. If the sampled spot contains an immune cell, the following events model anti-HIV immune expansion and contraction. Firstly, the immune cells can attempt to divide with a probability given by: *C x number of productively infected cells/N,* which is set to unity if the expression is greater than one. Hence, the rate of immune cell expansion is proportional to the number of productively infected cells that are present. A randomly chosen target spot is selected for the daughter immune cell, and if this spot is empty, the daughter immune cell is placed there. If the target spot is already occupied, cell division is aborted. With a probability H, the immune cell dies. In this way the immune cell population expands in proportion to the number of productively infected cells and declines as the productively infected cells decline. The immune cells can inhibit the virus population in the following way: When an infected cell is chosen for an

infection event, there is a probability, determined by the number of immune cells, that the infection event is aborted. This probability is given by *(F x number of immune cells)/N*, which is again set to unity if this expression exceeds one. This corresponds to non-lytic immune activity, and we will concentrate on this mode in our analysis. Lytic activity that kills infected cells can be incorporated in a similar manner, but we do not focus on this here. ODE modeling work described in the main text has demonstrated that results are similar for both modes of immune activity.

6. Application of model to replacement of wild-type with escape mutants in latent reservoir

The agent-based model discussed in the main text suggests that an increase in virus load during HIV infection can lead to reduced viral archiving in the latent reservoir. This can explain the observation that CTL escape leads to replacement of the sensitive virus with CTL escape mutants in the latent reservoir [15]. To demonstrate this more specifically, we modified our agent-based model to track two hypothetical virus strains and consider the immune cells in the simulation to be CTL. One virus strain is assumed to be susceptible to immune attack (larger values of C and F), and the second strain is assumed to be less susceptible to immune attack (smaller values of C and F). This escape strain is also assumed to have a certain replicative fitness cost compared to the wild-type [16]. In this setting, the two virus strains compete for the target cell population. Furthermore, when more than one productive virus is present in the same cell, the probability to pick a given provirus for reproduction and infection is proportional to the fraction of this provirus in the cell. The competition dynamics in such a model have been studied recently, and the outcome is typically the survival of the faster replicating virus, and exclusion of

the inferior virus strain [17]. The simulation is started with only the susceptible strain present, which leads to stochastic fluctuations around a relatively low equilibrium number of infected cells, because the infection is controlled by the ongoing immune response (Figure S1). During

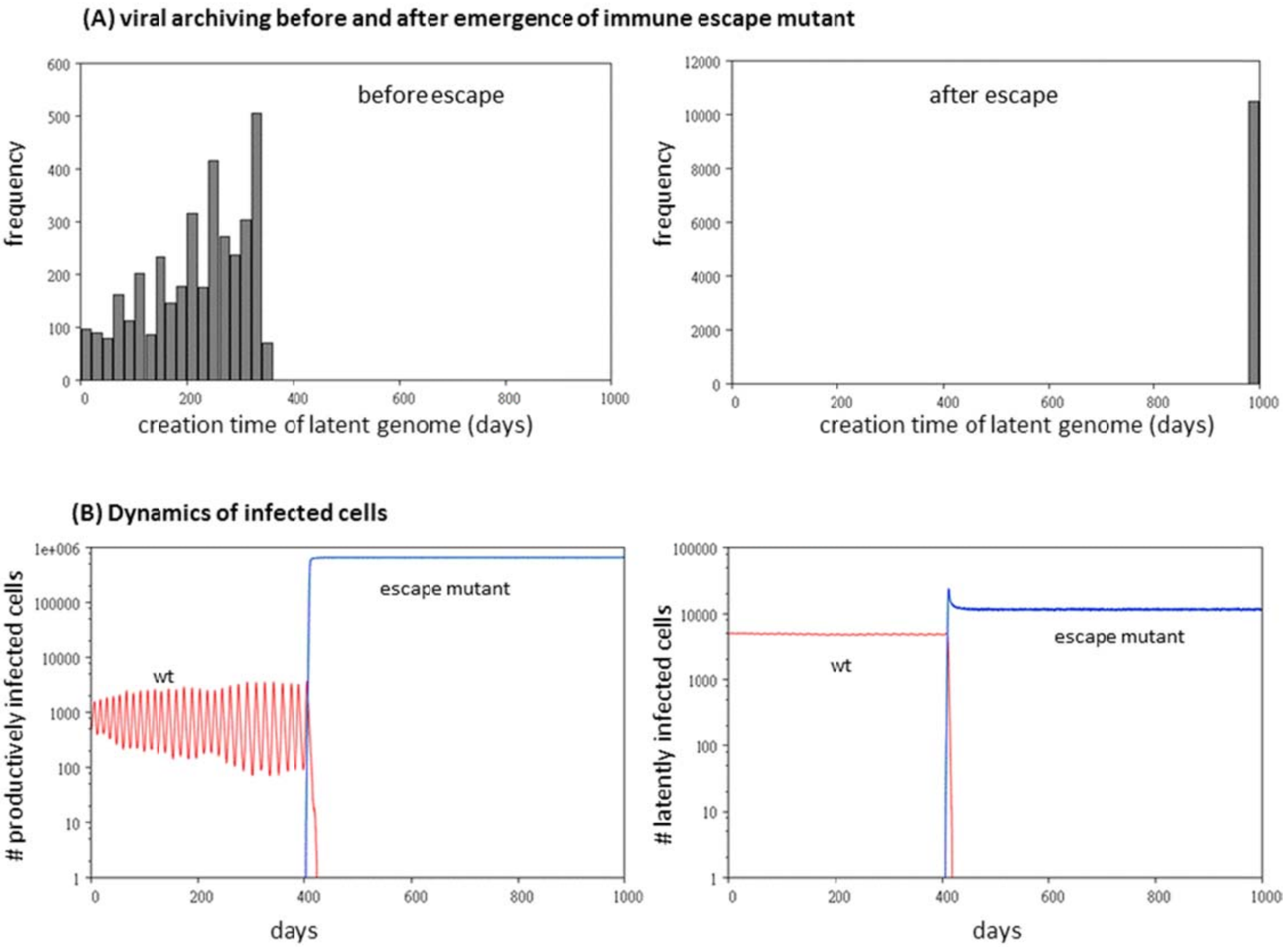

infection, however, the susceptible strain is allowed to mutate to give rise to the resistant strain. As shown in Figure S1A, a relatively large amount of archiving occurs before the virus escapes the immune response. Following the escape, however, the mutant takes over the virus population as well as the latent reservoir (Figure S1B), as observed in the clinical data [15]. Consequently, the degree of archiving becomes significantly less (Figure S1A).

Figure S1. *Agent based model with two virus strains: a CTL-sensitive strain and a CTL escape strain. The simulation is started around equilibrium values with only wild-type virus present and without mutations allowed, and runs like this up to time step 400 days. At this time, mutation processes were switched on in the simulation, and this could result in the generation of the escape mutant. (A) These graphs plot the frequency histogram of the creation time of all latent viral genomes present at day 350 (before mutations are switched on) and at day 1000, after the escape mutant has been generated. The latent genomes were counted only in cells that did not also contain a productive virus. A stronger immune response is assumed. Hence, extensive archiving is observed before immune escape, but almost no archiving after immune escape. (B) Time series showing the development of the productively and latently infected cells over time. After mutations are switched on, the escape mutant emerges readily and grows to fixation, resulting in a higher number of infected cells. Once escape occurs, the CTL-sensitive virus also quickly disappears from the latent reservoir and is replaced with the CTL escape mutant. Parameters were the same as in Figure 3 with the longer half-life of latently infected cells. The immune escape mutant was assumed to have a 20% replicative cost, and was assumed to be only partially inhibited by immunity, such that $F_{mutant} = F_{wild\text{-}ytpe}/5$. The immune escape mutant was assumed not to stimulate the immune response. The mutation rate $\mu=10^{-3}$ was assumed to be relatively large for the purpose of this simulation, as the population sizes are kept relatively small for the sake of computational speed.*

References

1. Levy D.N., Aldrovandi G.M., Kutsch O., Shaw G.M. 2004 Dynamics of HIV-1 recombination in its natural target cells. *Proceedings of the National Academy of Sciences of the United States of America* **101**(12), 4204-4209.
2. Gelderblom H.C., Vatakis D.N., Burke S.A., Lawrie S.D., Bristol G.C., Levy D.N. 2008 Viral complementation allows HIV-1 replication without integration. *Retrovirology* **5**, 60.

3. Bregnard C., Pacini G., Danos O., Basmaciogullari S. 2012 Suboptimal provirus expression explains apparent nonrandom cell coinfection with HIV-1. *Journal of virology* **86**(16), 8810-8820.
4. Perelson A.S., Neumann A.U., Markowitz M., Leonard J.M., Ho D.D. 1996 Hiv-1 Dynamics in-Vivo - Virion Clearance Rate, Infected Cell Life- Span, and Viral Generation Time. *Science* **271**(5255), 1582-1586.
5. Ramratnam B., Mittler J.E., Zhang L., Boden D., Hurley A., Fang F., Macken C.A., Perelson A.S., Markowitz M., Ho D.D. 2000 The decay of the latent reservoir of replication-competent HIV-1 is inversely correlated with the extent of residual viral replication during prolonged anti-retroviral therapy. *Nature medicine* **6**(1), 82-85.
6. Siliciano J.D., Kajdas J., Finzi D., Quinn T.C., Chadwick K., Margolick J.B., Kovacs C., Gange S.J., Siliciano R.F. 2003 Long-term follow-up studies confirm the stability of the latent reservoir for HIV-1 in resting CD4+ T cells. *Nature medicine* **9**(6), 727-728.
7. Ribeiro R.M., Qin L., Chavez L.L., Li D., Self S.G., Perelson A.S. 2010 Estimation of the initial viral growth rate and basic reproductive number during acute HIV-1 infection. *Journal of virology* **84**(12), 6096-6102.
8. Chavez L., Calvanese V., Verdin E. 2015 HIV Latency Is Established Directly and Early in Both Resting and Activated Primary CD4 T Cells. *PLoS pathogens* **11**(6), e1004955.
9. Komarova N.L., Anghelina D., Voznesensky I., Trinite B., Levy D.N., Wodarz D. 2013 Relative contribution of free-virus and synaptic transmission to the spread of HIV-1 through target cell populations. *Biology letters* **9**(1), 20121049.
10. Chen P., Hubner W., Spinelli M.A., Chen B.K. 2007 Predominant mode of human immunodeficiency virus transfer between T cells is mediated by sustained Env-dependent neutralization-resistant virological synapses. *Journal of virology* **81**(22), 12582-12595.
11. Hubner W., McNerney G.P., Chen P., Dale B.M., Gordon R.E., Chuang F.Y., Li X.D., Asmuth D.M., Huser T., Chen B.K. 2009 Quantitative 3D video microscopy of HIV transfer across T cell virological synapses. *Science* **323**(5922), 1743-1747.
12. Jung A., Maier R., Vartanian J.P., Bocharov G., Jung V., Fischer U., Meese E., Wain-Hobson S., Meyerhans A. 2002 Multiply infected spleen cells in HIV patients. *Nature* **418**(6894), 144.
13. Josefsson L., King M.S., Makitalo B., Brannstrom J., Shao W., Maldarelli F., Kearney M.F., Hu W.S., Chen J., Gaines H., et al. 2011 Majority of CD4+ T cells from peripheral blood of HIV-1-infected individuals contain only one HIV DNA molecule. *Proceedings of the National Academy of Sciences of the United States of America* **108**(27), 11199-11204.
14. Josefsson L., Palmer S., Faria N.R., Lemey P., Casazza J., Ambrozak D., Kearney M., Shao W., Kottilil S., Sneller M., et al. 2013 Single cell analysis of lymph node tissue from HIV-1 infected patients reveals that the majority of CD4+ T-cells contain one HIV-1 DNA molecule. *PLoS pathogens* **9**(6), e1003432.
15. Deng K., Pertea M., Rongvaux A., Wang L., Durand C.M., Ghiaur G., Lai J., McHugh H.L., Hao H., Zhang H., et al. 2015 Broad CTL response is required to clear latent HIV-1 due to dominance of escape mutations. *Nature* **517**(7534), 381-385.
16. Ganusov V.V., Goonetilleke N., Liu M.K., Ferrari G., Shaw G.M., McMichael A.J., Borrow P., Korber B.T., Perelson A.S. 2011 Fitness costs and diversity of the cytotoxic T lymphocyte (CTL) response determine the rate of CTL escape during acute and chronic phases of HIV infection. *Journal of virology* **85**(20), 10518-10528.
17. Phan D., Wodarz D. 2015 Modeling multiple infection of cells by viruses: Challenges and insights. *Mathematical biosciences* **264**, 21-28.
